# Atypical Vesicular Skin Eruption Following a Low-Dose Methotrexate Dosing Error: A Case Report

**DOI:** 10.7759/cureus.110259

**Published:** 2026-06-04

**Authors:** Mary Grace H London, Kelly M Frasier, Nicole K Werpachowski, James Scaduto

**Affiliations:** 1 Dermatology, Edward Via College of Osteopathic Medicine, Auburn, USA; 2 Dermatology, Northwell Health, New Hyde Park, USA; 3 Department of Medicine, Lenox Hill Hospital, Northwell, New York, USA; 4 Internal Medicine, Northern Dutchess Hospital, Rhinebeck, USA

**Keywords:** drug-related side effects and adverse reactions, leucovorin, low-dose methotrexate, methotrexate toxicity, oral mucositis

## Abstract

Methotrexate is widely used to manage various autoimmune conditions, including psoriasis and psoriatic arthritis. Although weekly low-dose regimens are generally well-tolerated, dosing errors, particularly daily instead of weekly administration, can result in acute methotrexate toxicity with mucocutaneous and hematologic complications.

We present a case of clinically significant methotrexate toxicity manifesting with painful oral mucositis and vesicular cutaneous lesions on the right lateral abdomen and right supraclavicular region in a 68-year-old Caucasian female patient with psoriasis and psoriatic arthritis after inadvertently taking 15 mg of methotrexate daily for five consecutive days (75 mg cumulative) instead of her prescribed weekly 15 mg dose. The patient was admitted for intravenous hydration with sodium bicarbonate and leucovorin 200 mg every six hours, then transitioned to oral leucovorin for an additional 48 hours on discharge. Mucosal symptoms gradually resolved during her four-day admission, no new skin lesions developed after hospital day 2, and laboratory values trended toward normalization without clinically significant myelosuppression, neutropenia, thrombocytopenia, or hepatorenal injury.

This case illustrates that an inadvertent daily-instead-of-weekly dosing error can produce clinically significant mucocutaneous toxicity, and that prompt recognition with leucovorin rescue may help limit progression to the myelosuppression and systemic complications that can accompany methotrexate toxicity. It is important for clinicians to remain vigilant for toxic adverse effects to prevent progression to life-threatening complications and enhance patient education to avoid dosing errors.

## Introduction

Methotrexate, a mainstay treatment for autoimmune conditions like psoriasis and psoriatic arthritis, has a narrow therapeutic index, requiring precise dosing and monitoring. Although chemotherapy protocols may require doses as high as 12,000 mg/m^2^, dermatologic indications typically involve lower doses, beginning at 7.5-15 mg and titrated to 30 mg weekly based on patient response [1,2]. Among the known adverse effects at low doses, mucocutaneous toxicity, including oral stomatitis and vesicular skin eruptions, is infrequent but significant. Stomatitis has been reported in approximately 5%-8% of patients receiving low-dose methotrexate [3]. These effects result from methotrexate’s inhibition of dihydrofolate reductase (DHFR) and other folate-dependent enzymes, which disrupt purine and thymidylate metabolism and impair DNA synthesis and repair in rapidly dividing epithelial cells, particularly those of the oral mucosa and cutaneous epithelium [4].

Methotrexate dosing errors in clinical practice are often linked to patient misunderstanding of weekly versus daily regimens. Methotrexate toxicity from dosing errors, even from small deviations from prescribed weekly doses, can be overlooked because severe adverse effects are commonly associated with high-dose chemotherapy regimens. Histologically, mucocutaneous toxicity is characterized by keratinocyte dystrophy resulting from impaired keratinocyte turnover, thereby compromising epithelial integrity and rendering the epithelium susceptible to mucocutaneous complications [5]. While these complications are recognized manifestations of methotrexate toxicity, their occurrence following an accidental dosing error highlights the importance of recognizing atypical presentations early and intervening promptly to reduce the risk of progression to systemic toxicity. Early recognition is critical to initiate leucovorin rescue therapy and prevent progression to systemic complications like myelosuppression.

## Case presentation

**Figure 1:**
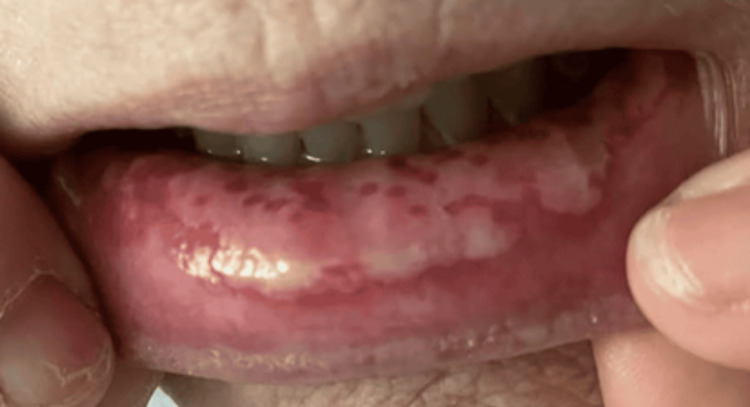
Methotrexate toxicity presentation in the oral mucosa Erythematous, eroded, and ulcerated oral mucosa without bleeding, photographed on hospital admission

A 68-year-old Caucasian female patient presented to the emergency department with persistent nausea, emesis, and painful oral ulcers. Past medical history included psoriasis, psoriatic arthritis, chronic deep vein thrombosis on warfarin, and hypertension. She had been on weekly methotrexate 15 mg orally for the preceding few months for the management of her psoriasis and psoriatic arthritis, followed by rheumatology. She was not on concurrent folic acid supplementation and was also taking pro re nata (PRN) naproxen sodium, which was discontinued on admission. She reported mistakenly taking methotrexate 15 mg daily for five consecutive days (75 mg total) instead of the prescribed weekly dose. Adverse effects began on day 6 with gastrointestinal discomfort and oral mucositis. She denied systemic symptoms, including fever, chills, headaches, vision changes, chest pain, dyspnea, easy bruising, or bleeding. Vital signs were stable. On initial examination, the oral mucosa was erythematous and ulcerated without bleeding (Figure 1). Laboratory workup included a complete blood count (CBC), a comprehensive metabolic panel (CMP), an international normalized ratio (INR), and a serum methotrexate level. Alternative etiologies for the mucocutaneous findings, including herpes simplex virus (HSV) stomatitis, contact dermatitis, and infectious or allergic mucositis, were considered but felt unlikely given the clear temporal relationship to the dosing error, the absence of viral prodrome or grouped vesicular distribution typical of HSV, and the lack of fever or systemic infectious symptoms. Toxicology consultation with the regional Poison Control Center confirmed acute methotrexate toxicity and advised admission.

**Figure 2:**
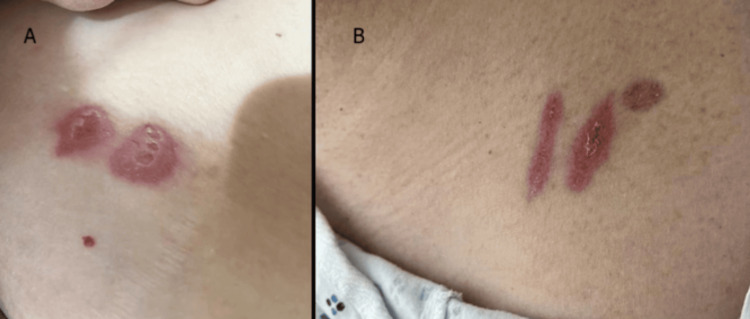
Methotrexate toxicity presenting with resolving cutaneous lesions Multiple vesicles and superficial erosions overlying sharply demarcated erythematous patches on the right lateral abdomen (A) and right supraclavicular region (B) during hospital admission. The lesions emerged on hospital day 2 and were already resolving at the time the photographs were obtained

Inpatient management included intravenous (IV) hydration with sodium bicarbonate (to alkalinize urine and promote methotrexate excretion), serial monitoring of renal function and electrolytes, and leucovorin 200 mg IV every six hours, as directed by the regional Poison Control Center. Reported leucovorin rescue regimens for methotrexate toxicity vary widely, ranging from approximately 10 mg/m^2^ every six hours to higher doses used when there is concern for significant cumulative exposure or impaired clearance [6]. The regimen here was selected in consultation with toxicology based on the clinical severity of presentation. On hospital day 2, the patient developed new vesicular cutaneous lesions on the right lateral abdomen and right supraclavicular region (Figures 2A, 2B), consistent with epithelial dysfunction in the setting of methotrexate toxicity. The lesions were already in a resolving phase by the time photographs were obtained during admission.

Serum methotrexate level at the time of testing was <0.05 µmol/L. The transient decline in hemoglobin from 13.3 to 11.6 g/dL between days 1 and 2 may have reflected hemodilution from IV fluid resuscitation; there was no clinical evidence of bleeding or hemolysis, and values stabilized at 11.4-12.0 g/dL thereafter without a pattern suggestive of methotrexate-induced myelosuppression (Table 1). Although the admission INR of 1.7 exceeded the laboratory reference range, it was subtherapeutic for this patient, given her chronic warfarin therapy for deep vein thrombosis (target INR of 2.0-3.0), a fluctuation she reported as a recurrent and self-managed occurrence. The modest INR elevation throughout admission was therefore attributable to baseline warfarin rather than methotrexate-induced coagulopathy, and no bleeding was observed. The absolute neutrophil count remained within normal limits throughout hospitalization, with a nadir of 2.12 x 10^3^/uL on day 3, and no neutropenia developed. Oral mucositis was managed with magic mouthwash, allowing adequate pain relief for a soft diet. Throughout admission, mucosal symptoms gradually resolved, no new skin lesions developed after hospital day 2, and repeat labs revealed a trend toward improvement without evidence of clinically significant myelosuppression, hepatic/renal impairment, or systemic toxicity (Table 1).

**Table 1:** Laboratory values during hospitalization for methotrexate toxicity Laboratory trends were notable for mild, transient elevations in BUN and creatinine that normalized by day 3, without progression to acute kidney injury; a transient mild decline in white blood cell count (nadir 3.0 × 10^3^/uL on day 3) with a concurrent downward but in-range platelet trend. Neither reached clinically significant myelosuppression, and both were trending upward by day 4; INR and PT values were consistent with baseline warfarin therapy. Overall, the trends demonstrate clinical improvement with supportive care without progression to clinically significant hematologic or hepatorenal toxicity ^*^Below the normal reference range ^**^Above the normal reference range WBC: white blood cell, BUN: blood urea nitrogen, INR: international normalized ratio, PT: prothrombin time

Parameter	Reference range	Day 1 (on admission)	Day 2	Day 3	Day 4
WBC count (×10^3 ^u/L)	3.5-11.5	7.6	3.6	3.0^*^	4.6
Hemoglobin (g/dL)	12.0-15.1	13.3	11.6^*^	11.4^*^	12.0
Platelet (×10^3 ^u/L)	150-450	306	208	177	178
BUN (mg/dL)	7-20	25^**^	16	13	-
Creatinine (mg/dL)	0.6-1.0	1.09^**^	0.95	0.95	-
INR	0.8-1.2	1.7^**^	1.8^**^	1.5^**^	1.4^**^
PT (seconds)	10-13	20.4^**^	20.8^**^	17.7^**^	16.6^**^

Upon discharge, the patient was clinically stable and transitioned to oral leucovorin 200 mg every six hours for 48 hours. Follow-up was arranged for labs (CBC, CMP, and serum methotrexate) and rheumatology evaluation to reassess disease-modifying therapy. Methotrexate was held indefinitely. Poison control remained involved in the patient’s postdischarge care.

## Discussion

The management of methotrexate toxicity resulting from low-dose dosing errors is complex and multifaceted, as repeated administration in place of the prescribed weekly regimen can rapidly produce clinically significant toxicity. Although the individual dose in this case (15 mg) represents a standard therapeutic low dose for autoimmune indications at which toxicity is uncommon, five consecutive daily doses produced a supratherapeutic cumulative exposure with clinically significant mucocutaneous manifestations. Methotrexate primarily affects tissues with rapid cell turnover, including the oral mucosa, skin, and bone marrow. By impairing keratinocyte turnover, it weakens the integrity of the skin and oral mucosa, resulting in the keratinocyte dystrophy seen histologically in methotrexate-induced skin toxicity [5]. In our patient, these changes account for 1) the oral mucositis and 2) the vesicular cutaneous lesions. The bone marrow, by contrast, was largely spared, without clinically significant myelosuppression, likely reflecting the limited cumulative exposure and early initiation of treatment.

The patient’s undetectable serum methotrexate level (<0.05 µmol/L) at admission, in the setting of clinically evident toxicity, reflects methotrexate's pharmacokinetic profile. After cellular uptake, the drug is converted to methotrexate polyglutamates, which are retained intracellularly and continue to inhibit DHFR and other folate-dependent enzymes after the parent compound has cleared from serum [4,7,8]. In published series of low-dose methotrexate toxicity, serum levels have not been shown to reliably correlate with the degree of clinical or laboratory toxicity [7,8].

Several case reports and series describe this risk due to dosing errors at low weekly doses. One report described a 63-year-old female patient with severe mucositis and hematologic abnormalities after incorrectly ingesting 10 mg daily, instead of weekly, while another reported a 69-year-old man with rheumatoid arthritis who developed oral and lip ulcerations after taking 7.5 mg twice daily for five days [9,10]. Asaduzzaman et al. described a 35-year-old female patient who developed multisystem toxicity after taking 10 mg daily for 12 days [4]. Larger series of low dose methotrexate toxicity have reported substantial morbidity and mortality: Janet et al. described 19 patients with inadvertent daily methotrexate ingestion, with three deaths attributed to delays in medical care [3]; Subedi et al. described a five-patient series with a mortality in 2 of 5 [11]; and Kivity et al., in a cohort of 28 patients hospitalized for low-dose methotrexate toxicity, found pancytopenia in 78% and mucositis in 53%, with overall mortality of 25%, primarily attributable progression to pancytopenia followed by sepsis [8]. Fatal outcomes have been reported even in patients receiving chronic low-dose therapy, particularly in those with end-stage renal disease or a lack of folate supplementation [7]. Concurrent nonsteroidal anti-inflammatory drug (NSAID) use is similarly recognized as a risk factor, as NSAIDs can reduce methotrexate renal clearance; in the present patient, PRN naproxen was being taken at the time of the dosing error and was discontinued on admission.

A recent multicenter retrospective cohort of 54 patients with oral methotrexate therapeutic errors reported organ system dysfunction in 57% of patients, with mucositis the most common manifestation (94% of those affected) and dermatologic manifestations in 58%; no patient in that cohort developed organ system dysfunction at cumulative doses below 37.5 mg or fewer than three consecutive days of dosing [12]. The present patient’s cumulative dose of 75 mg over five consecutive days exceeded both reported thresholds, consistent with the development of clinically significant mucocutaneous toxicity in this case. While mucocutaneous toxicity is a recognized manifestation of methotrexate overdose, the dermatologic manifestations most commonly reported include dermatitis, ulceration, epidermal necrosis, and alopecia [12]; the discrete vesicular morphology observed in the present patient is less commonly reported in the literature [6,11]. Most reported cases of methotrexate toxicity involve cumulative doses comparable to or higher than those in the present patient, for instance, the range of 35-150 mg administered over several consecutive days in the psoriasis dosing-error literature [13]. Thus, this case is noteworthy not for the cumulative dose itself, which is comparable to that in other reported cases of dosing-error toxicity. Instead, it is important to recognize mucocutaneous toxicity early and administer prompt leucovorin rescue, which may help prevent progression to the hematologic and systemic complications that frequently accompany methotrexate toxicity.

One of the challenges in recognizing methotrexate toxicity is the nonspecific nature of early symptoms, often misattributed to unrelated causes. In particular, oral stomatitis can be mistaken for HSV, while vesicular skin eruptions can resemble allergic reactions or contact dermatitis. For example, one case showed how daily, instead of weekly, low-dose methotrexate led to pancytopenia and oral ulcerations misdiagnosed as an infectious process [14]. Another case reported ceftriaxone initiation for presumed infection, leading to delays in treatment before discovering that the mucocutaneous symptoms were related to a methotrexate self-dosing error; symptoms resolved with leucovorin therapy [15]. Timely leucovorin administration is central to management, as it bypasses the blocked DHFR, facilitating cellular recovery. Hydration, urine alkalinization, and serial monitoring further aid in supportive management [16]. Together, these cases highlight the importance of both heightened clinical suspicion and prompt leucovorin rescue in patients on methotrexate presenting with new mucosal or cutaneous findings.

Preventing low-dose methotrexate toxicity relies on patient education and close follow-up, especially as dosing errors are not uncommon in polypharmacy. A study analyzing 106 cases of methotrexate toxicity found that dosing errors, often due to misunderstanding, were the most common cause, particularly in older adults with complex medication regimens [17]. Recent recommendations to reduce such errors include using blister packs or pill organizers, setting default weekly dosing in electronic prescribing systems, and requiring patients to repeat dosing instructions at the point of dispensing [12]. Clear verbal and written instructions, reinforcement counseling by pharmacists, and the use of pill organizers can help mitigate these errors and reinforce safer prescribing practices to optimize patient outcomes.

## Conclusions

While low-dose methotrexate is widely used in the management of autoimmune and oncologic conditions and is typically well-tolerated, this case illustrates that even a short period of inadvertent daily administration can result in clinically significant mucocutaneous toxicity. The potential for adverse effects, even when therapeutic individual doses are taken at the wrong frequency, highlights the need for meticulous prescribing, effective patient counseling, and early recognition of symptoms. It is important to be aware that an inadvertent series of daily doses, taken in error in place of the prescribed weekly dose, can produce clinically significant mucocutaneous toxicity, and that early recognition may allow timely leucovorin rescue to reduce the risk of progression to systemic involvement. Ultimately, this case reinforces the importance of balancing methotrexate’s clinical benefits with its risk for toxicity, emphasizing the need for heightened clinical awareness and thorough patient management.
